# Land-use Suitability is Not an Intrinsic Property of a Land Parcel

**DOI:** 10.1007/s00267-022-01764-y

**Published:** 2022-12-16

**Authors:** Ton Snelder, Linda Lilburne, Doug Booker, Amy Whitehead, Simon Harris, Scott Larned, Anette Semadeni-Davies, David Plew, Richard McDowell

**Affiliations:** 1 LWP Limited, PO Box 70, Lyttelton, New Zealand; 2 Landcare Research, PO Box 69040, Lincoln, 7640 New Zealand; 3 National Institute of Water and Atmospheric Research, PO Box 8602, Christchurch, 8011 New Zealand; 4 National Institute of Water and Atmospheric Research, Private Bag 99940, Auckland, New Zealand; 5 AgResearch, Lincoln Science Centre, Private Bag 4749, Christchurch, 8140 New Zealand; 6 Faculty of Agriculture and Life Sciences, Lincoln University, PO Box 84, Lincoln, Christchurch, 7647 New Zealand

**Keywords:** Contaminant loads, Environmental targets, Land and water management, Land use, Suitability

## Abstract

Agricultural production has economic, environmental, social and cultural consequences beyond farm boundaries, but information about these impacts is not readily available to decision makers. This study applied the land use suitability concept by carrying out an assessment of a region that has the potential for intensification of agricultural production, but where eutrophication of river and estuary receiving environments due to nitrogen enrichment is a significant issue. The assessment evaluated three indicators for each farmable land parcel in the region: productive potential (the inherent productive and economic potential of the parcel), relative contribution (the potential for the parcel to contribute nitrogen to receiving environments compared to other land parcels), and pressure (the load of nitrogen delivered to receiving environments compared to the loads that ensure environmental objectives are achieved). The assessment indicated that land with high suitability for land-use intensification in Southland is limited because areas with high productive potential and low relative contribution rarely coincide with receiving environments with low pressure. Existing data, methods and models can be used to calculate the indicators under different choices for regional land-use intensity and receiving environment objectives. However, the spatial resolution and accuracy that is achievable may preclude using assessment outputs to make land use decisions at small spatial scales such as individual farms. The study highlighted that land use suitability is not an intrinsic property of a land parcel because it is dependent on choices about land use elsewhere in the landscape and the environmental objectives, and that land use suitability is inherently subjective because of decisions that concern how indicators are combined and weighted.

## Introduction

The intensification of agricultural production to meet growing demands for food and economic well-being has the potential to degrade land, water, biodiversity and climate (Foley et al. 2011; Meyfroidt 2018). It is also increasingly recognized that land-use decisions have economic, environmental, social and cultural consequences beyond farm boundaries (Goldstein et al. 2012; Liebig et al. 2017). For example, intensification of agriculture can degrade water quality by increasing nutrient loads in downstream receiving environments, with adverse environmental and socio-economic consequences (e.g., Glibert et al. 2014). Despite the general recognition that land-use decisions have both beneficial and adverse consequences, integrated and location-specific information about the suitability of land for agricultural use is not readily available to land-use decision makers (Tian et al. 2018).

Building on work by Collins et al. (2001), McDowell et al. (2018) introduced the land use suitability (LUS) concept as a framework for assessing the suitability of land for agricultural production both within and beyond farm boundaries. LUS differs from traditional land suitability frameworks (e.g., Food and Agriculture Organization 1977; Halder 2013) that focus only on matching crop potential to the productive potential of land within farm boundaries. LUS recognizes that land use has economic, environmental, social and cultural impacts and that these impacts can accumulate in space and occur far from the individual farms where production occurs. The LUS approach provides stakeholders with indicators that convey information about both the productive potential of land and the cumulative impact of land use on other societal values.

McDowell et al. (2018) proposed a specific implementation of LUS where the suitability for agricultural production of each parcel of land that could be farmed within a landscape is considered in conjunction with environmental constraints that are defined by water-quality objectives for downstream receiving environments (e.g., aquifer, streams, rivers, estuaries). This implementation of LUS was referred to as sustained Productivity within Environmental Constraints (PEC). In the conceptual model developed by McDowell et al. (2018), PEC assessments for each land parcel across a landscape are carried out with respect to a specified contaminant (e.g., nitrogen or sediment) using three indicators: productive potential, relative contribution and pressure. Productive potential characterizes the inherent productive and economic potential of each land parcel and depends on local characteristics of the land and the desired (economic) objective. Relative contribution characterizes the potential for each land parcel to contribute contaminants, relative to other land parcels, to downstream receiving environments. Pressure characterizes the contaminant load delivered to a receiving environment compared to the load that ensures that environmental objectives are met. Similar indices composed of multiple socio-economic, agricultural and environmental indicators have been used to compare land-use scenarios (e.g., Gómez-Limón and Sanchez-Fernandez 2010; Parish et al. 2016).

Productive potential is an intrinsic and invariant property of each land parcel; its variation across a landscape can therefore be represented as a static map. However, values of the relative contribution and pressure indicators for each land parcel are influenced by land-use decisions made for multiple land parcels across a landscape as well as choices concerning environmental objectives. Therefore, in a PEC assessment, a model representing the land-water system is required to evaluate relative contribution and pressure based on scenarios that specify land use in the wider environment and environmental objectives. McDowell et al. (2018) proposed that the three indicators can be expressed categorically, mapped at catchment—national scales, and used to support decision making around environmental problems, making strategic land assessments and planning land development and investment. The indicators can be evaluated for a single contaminant, or for each of several contaminants (e.g., nitrogen, sediment load) and then combined into an LUS index, to facilitate judging the relative suitability of land parcels for land uses that result in contaminant losses.

This study applied the LUS concept by carrying out PEC assessments across a region that has the potential for intensification of agricultural production but where eutrophication of rivers and estuaries due to nutrient emissions from agriculture is a significant environmental issue (Environment Southland 2011). The study aim was to demonstrate the utility of the LUS concept using currently available data and models, and to highlight the role of subjective decisions and limits to accuracy that are likely to be unavoidable but important aspects of this type of land-use assessment.

## Study Area

**Fig. 1:**
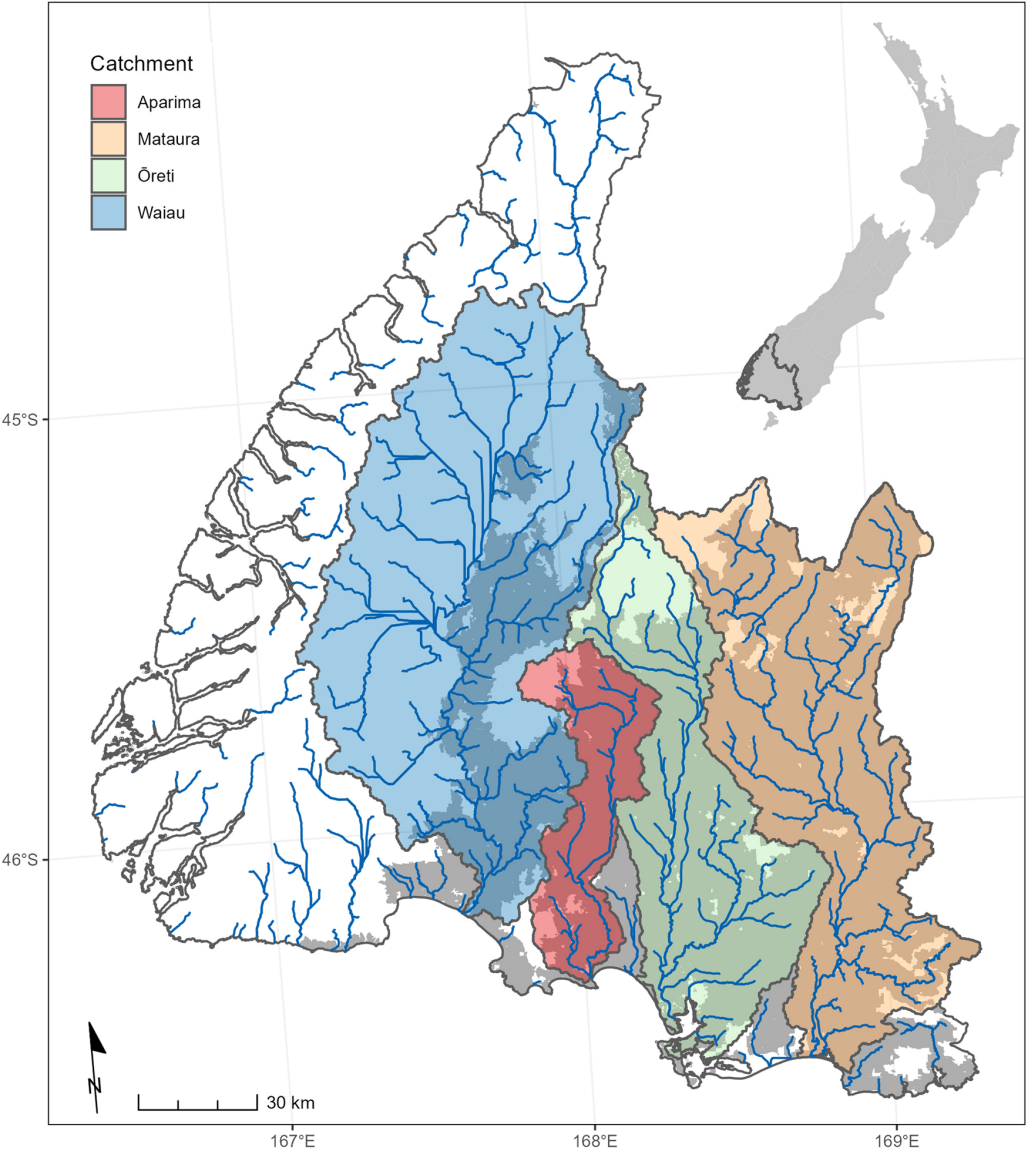
Map of the Southland region. The main catchment boundaries and mainstem rivers are shown. The dark shaded areas within each of the main catchments indicate land that is classified as farmable. The white and paler areas indicate conservation estate and land defined as not farmable. Note that Stewart Island to the south is part of the Southland region but is entirely conservation land and was therefore not included in the PEC assessment

The study area was part of the Southland region of New Zealand (Fig. 1). Southland is New Zealand’s southern most region and covers an area of ~2.9 million ha from the Tasman Sea on the west coast to the Pacific Ocean on the east coast, and Stewart Island to the south. The western and northern areas of Southland are dominated by mountainous terrain with altitudes above 2000 m. Central and southern Southland is dominated by gently sloping, low-elevation alluvial plains. The prevailing westerly airflow interacts with the regional topography to produce a strong west-east rainfall gradient. Average annual rainfall on the Southland plains varies between 750 and 1500 mm year^−1^ and is relatively evenly distributed throughout the year. Variation in Southland climate and geology has produced a wide range of soils; the most common types in farmed areas are inceptisols and entisols (Ledgard 2013).

**Fig. 2:**
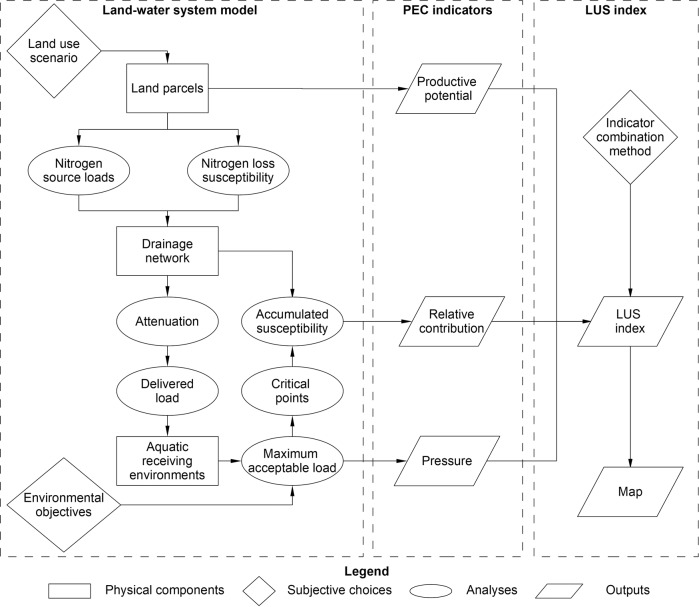
Schematic diagram of the PEC assessment process. The diagram defines the key model components that represent the land-water system, the analysis steps involved in calculating the three indicators and the combination of the indicators into a single LUS index

Public conservation land makes up 53% of the region, most of which is in Fiordland National Park and Rakiura National Park (Stewart Island). Land cover on the conservation land is dominated by indigenous forest, shrubland and wetlands. The study area excluded Fiordland National Park and Rakiura National Park and focused on land within the catchments of four major rivers (Waiau, Aparima, Ōreti and Mataura; Fig. 2). Land cover in these catchments is dominated by perennial pasture grassland, exotic forest and other agricultural cover types (Ledgard 2013).

Agricultural land use across New Zealand has been associated with poor water quality (Larned et al. 2016) and increased nutrient loads discharged to coastal environments (Snelder et al. 2018). Land-use intensification in the Southland region over the last 20 years has been associated with shifts from sheep and beef grazing to more profitable dairy farming (Beukes et al. 2011). Dairy cow numbers in the region increased from 200,000 in the 2000/01 milking-season to over 596,000 in 2020/21 (Dairy NZ and LIC 2021). This change in land use represents a significant increase in land-use intensity from low-input sheep and beef farming systems to more intensive and high-input dairy farming systems (Monaghan et al. 2021). Increased nitrogen concentrations and loads in Southland rivers have been linked to declining water quality (Hamill and McBride 2003; Monaghan et al. 2007), and consequent eutrophication of rivers and estuaries (Stevens et al. 2022). There remains significant potential for agricultural intensification (Kaye-Blake et al. 2019), which would almost certainly be associated with increased nitrogen loss (Vogeler et al. 2014; Vibart et al. 2015).

## Data and Methods

The PEC assessment process requires the evaluation of three indicators: productive potential, relative contribution and pressure for each farmable land parcel in the study area. The relative contribution and pressure indicators provide information pertaining to a land parcel’s contribution to the cumulative impacts of land use and require the development of a model of the land-water system to be evaluated (Fig. 2). The relative contribution and pressure indicators depend on assumptions concerning the land-use intensity across the study area and the stringency of the objectives for receiving environments that represent the environmental constraints. This section describes the data and methods that were used to evaluate the three indicators, including the models that were used to represent the land-water system. The data, methods and outputs pertain to spatial entities and therefore most inputs and all outputs of the assessment can be represented and mapped in a Geographic Information System (GIS).

### Land Parcels

Land parcels (Fig. 1) are the fundamental spatial units for which the PEC indicators are evaluated. We defined land parcels as contiguous areas with relatively homogenous characteristics relevant to farm production and nutrient loss based on the intersection of three geospatial data layers. First, all land in the study area was assigned to one of three land cover categories (agricultural, urban and natural) derived from the national land cover database version 4 (LCDB4; lris.scinfo.org.nz), which differentiates 33 land-cover categories based on the analysis of Satellite Pour l'Observation de la Terre (SPOT-5) imagery from 2012 (see Supplementary Material for details). Second, individual farms were identified using polygons in AgriBase™ GIS (Asure Quality 2012). We intersected these two layers and classified as “farmable” all land that was either dominated by the agricultural land cover category as defined by LCDB4 or occupied by an existing farm as defined by AgriBase. Finally, we intersected the combination of the above layers with sub-catchment boundaries defined by a GIS-based digital representation of the surface water drainage network (hereafter, drainage network; Snelder and Biggs 2002). This drainage network was derived from 1:50,000 scale contour maps; it represents the study area’s rivers as 43,000 segments (delineated by upstream and downstream confluences), each of which is associated with its own sub-catchment. The sub-catchments in Southland have an average area of 66 ha. The intersected data layers yielded a total of 61,411 farmable parcels in the study area with an average area of 21 ha.

A land use capability (LUC) category from the Land Resource Information (LRI) geospatial layer (Landcare Research 2018) was assigned to each land parcel. The LUC system provides an indication of the productive versatility of land parcels for a range of land uses, and identifies key constraints such as erosion (Lynn et al. 2009). Spatial variation in productive versatility is represented in the LUC system using eight ordinal categories where the limitations to productive use increase, and the versatility of use decreases, from LUC class 1 to 8. Classes 1 to 4 are classified as suitable for arable cropping or intensive pastoral grazing while LUC classes 5 to 7 are more suited to extensive pastoral farming or forestry, and class 8 is usually non-productive. Each land parcel in the region was assigned the spatially dominant LUC category for that parcel. Because land parcels were small, only one LUC category was present in the majority (80%) of parcels and 18% had two adjacent LUC categories.

**Fig. 3:**
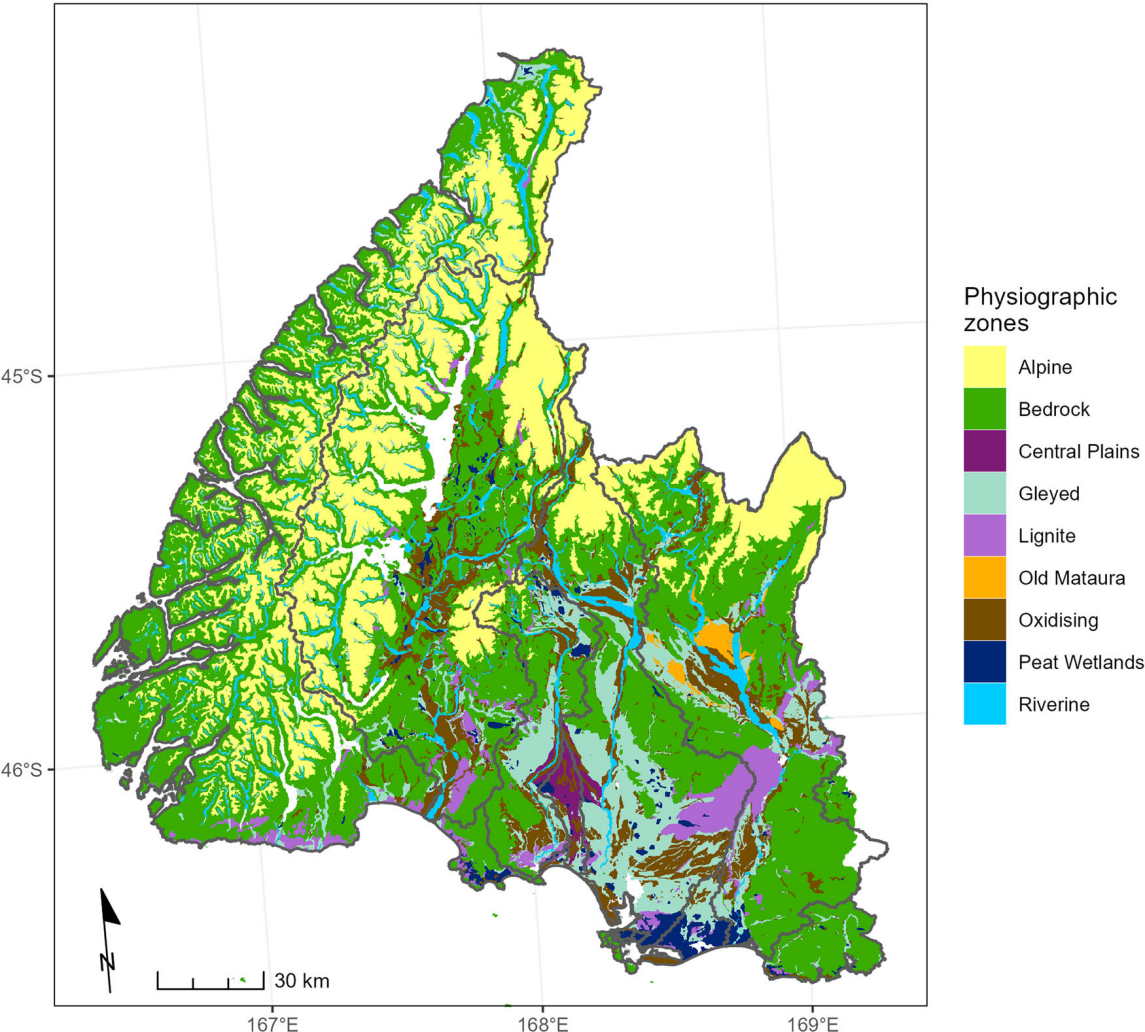
Map of the physiographic classification system of Hughes et al. (2016). The location of the variants of physiographic zones are not shown. See Table 1 for details of each physiographic zone

We also assigned each land parcel to a class defined by a physiographic classification system (Hughes et al. 2016). The classification comprised nine physiographic zones (Fig. 3) and eight sub‐zones (referred to as variants). These zones discriminate variation in the susceptibility to contaminant loss of land based on consideration of how landscape factors (topography, geology and soils) influence processes including contaminant mobilization, dilution, filtration, sorption, storage, transport and attenuation within the soil and underlying groundwater. As for the LUC assignments, each land parcel was assigned to the spatially dominant physiographic zone for that parcel.

### Drainage Network

Each segment of the drainage network is associated with attributes that describe connectivity between segments that collectively define the flow paths downstream of all land parcels. The drainage network delineated 241 unique sea-draining catchments within the study area, of which 166 contain farmable land parcels. The downstream end of each sea-draining catchment was defined by a terminal segment at the coast or an estuary.

Each network segment was associated with several descriptive characteristics. The position of the segment in the drainage network was described by stream order (Strahler 1964). Each segment was also associated with predicted median total nitrogen (TN) concentration based on Snelder et al. (2018); see Supplementary Material for details.

### Aquatic Receiving Environments

Receiving environments (Fig. 1) are locations in the drainage network to which nitrogen is delivered from upstream land and in which environmental constraints apply. Although there are some large lakes in the study area, only a small proportion have farmable land in their upstream catchments. Therefore, for this assessment, we defined two types of receiving environment, river segments and estuaries.

In principle, all segments of the drainage network are receiving environments. To reduce the computational burden, we limited the assessed river receiving environments to a representative sample of 2811 segments comprising segments of stream order three or higher, at which stream order increased (compared to segments immediately upstream), plus terminal segments (i.e., river mouths). Estuarine receiving environments were identified with the national delineation of estuaries and an associated estuary class (Hume et al. 2007). Eight significant coastal estuaries were defined by a geospatial layer (https://data.mfe.govt.nz/layer/3565-nz-coastal-hydrosystems/). We represented the connection of these estuaries to their catchments and all upstream land parcels by intersecting the estuary polygons with the terminal segments of the drainage network.

### Nitrogen Source Loads

We estimated TN source loads (i.e., lost from land parcels; Fig. 1) under two land-use scenarios: current land use and maximum pastoral land use. Nitrogen loss from farms was estimated using the nutrient balance model OVERSEER (Wheeler et al. 2014). In OVERSEER, the proportion of nitrogen excreted by livestock is derived from a balance between animal intake, maintenance needs and removal of animal products from the farm. OVERSEER provides an estimate of nitrogen lost at the boundaries of farm land parcels which are defined in the vertical direction by the soil root zone and in the horizontal direction by the property boundary.

The first step in estimating farm losses was to assign each farm in the study area to a representative farm type based on combinations of four factors: (1) enterprise type (dairy, sheep and beef, forestry); (2) land-use intensity level (high, medium, low); (3) LUC category (1–8); and (4) drainage type (well drained, poorly drained). Each farm polygon defined by AgriBase^TM^ was assigned to the dominant LUC category and drainage class as described by the LRI geospatial layer. The current enterprise type and land use intensity were obtained from AgriBase^TM^. The combination of categories across the four factors resulted in 121 different farm types.

The OVERSEER model was then used to estimate rates of nitrogen loss (kg TN ha^−1^ year^−1^) for each of the farm types (see Vibart et al. 2015 for details). These loss rates were used to estimate the source load of nitrogen lost to water from all farmable land parcels under current and maximum land-use scenarios. The maximum pastoral land-use scenario was represented by converting all current sheep and beef farms on LUC class 1–4 land to high intensity dairy farming and increasing intensity on all existing dairy farms to high. Both conversions are associated with increased loss of nitrogen to water (Monaghan et al. 2021).

Nitrogen loss rates for non-farm land were estimated using the catchment contaminant model SPARROW (Spatial Regional Regression on Watershed attributes; Alexander et al. 2004). SPARROW is a hybrid mechanistic-regression catchment model that routes contaminant loads generated by all land parcels down the drainage network. The SPARROW model was based on the digital drainage network and, at each receiving environment, mass budgeting was used to represent a long-term equilibrium condition, expressed at an annual time scale. For this study, a recent implementation of SPARROW for the Southland region was used (Elliott et al. 2005). Inputs to the SPARROW model were loads of nitrogen generated by farmable land parcels from the OVERSEER modeling, and loads associated with 11 regionally significant point sources (i.e., treated wastewater discharges). The loads generated by all non-farm land parcels were estimated directly by SPARROW. Model calibration involved reconciliation of the annual mass balance with observed instream loads calculated from flow and nitrogen concentration observations at 27 monitoring sites in the region. The loads from all non-farm land parcels and point sources were constant for the two land-use scenarios.

### Nitrogen Loss Susceptibility

The susceptibility of each farmable land parcel to nitrogen loss was quantified based on the physiographic classification system (Fig. 3). The nine physiographic zones are defined by unique combinations of landscape factors (topography, geology and soils) that discriminate variation in the susceptibility to loss of nitrogen and other contaminants (Hughes et al. 2016). These landscape factors account for regional variation in processes that determine nitrogen loss susceptibility including the attenuation of nitrogen in soil and groundwater and hydrological processes that determine the retention and transport of water.

The attenuation of nitrogen in soil and groundwater is a microbially mediated chemical reaction that converts the nitrate ion to nitrogen dioxide gas, which is lost to the atmosphere (i.e., denitrification, Seitzinger et al. 2006; Tratnyek et al. 2011). Denitrification requires certain conditions in soil and groundwater including low oxygen, an abundance of microbially metabolizable electron donors (e.g., organic carbon, ferrous iron) and retention in water for sufficient time for denitrification to occur (Bartlett and James 1993; Wilson et al. 2018). The potential for denitrification in soil and groundwater varies spatially depending on the extent to which these required conditions occur (Vepraskas and Faulkner 2001; McMahon and Chapelle 2008). When there is appreciable attenuation, the risk to downstream receiving environments is decreased.

The potential for attenuation of nitrogen is also controlled by the transport pathway taken by water discharged from at the farm boundary. Hughes et al. (2016) distinguish three pathways that transport nitrogen from land parcels to receiving environments: (1) vertical transport via soils to the groundwater system and then to streams and rivers; (2) horizontal transport through the soil zone (i.e., interflow); and (3) horizontal transport across the land surface by overland flow. The dominance of these transport processes varies in Southland due to differences in soils and topography, leading to corresponding variation in the potential for retention and attenuation of nitrogen. Deep and porous soils have greater potential to retain nitrogen that can be used by plants. Nitrogen in shallow porous soils on the other hand may be lost vertically below the root zone. Attenuation in this case is dependent on the groundwater characteristics. Where soils are poorly drained and/or on sloping land, horizontal transport by overland flow tends to be dominant and there is limited nitrogen attenuation. Variants of some physiographic zones identify areas where horizontal transport via overland flow or via artificial drainage (e.g., tile drains) becomes dominant when soils are saturated. These variants have higher susceptibility to nitrogen loss than their non-variant counterparts because denitrifying zones in soils and groundwaters are temporarily by-passed entirely (overland flow) or partially (artificial drainage).

Nitrogen loss susceptibility for farms were based on their assigned physiographic zone and variant, each of which is associated with one of five nitrogen loss susceptibility categories: low, moderate, high, very high and extremely high (Table 1). These categorizations were based on assessment of the dominant transport pathway and denitrification potential of the soil and groundwater by Hughes et al. (2016). So that nitrogen loss susceptibility could be used to calculate the relative contribution index in subsequent steps, we assigned each category to a value between 0 and 1 based on expert knowledge of the dominant transport pathways and potential for attenuation associated with each physiographic zone and associated variants. We refer to these values as the nitrogen loss susceptibility index (Table 1).

**Table 1:** Nitrogen loss susceptibility categories indices for land parcels in each physiographic zone (Fig. 3)

Physiographic class	Characteristics influencing retention of nitrogen and potential for attenuation	Nitrogen loss susceptibility category	Nitrogen loss susceptibility index
Alpine	Steeply sloping land, shallow soils, with negligible retention and attenuation potential. Largely surface water transport.	Extremely High	0.9
Bedrock/Hill Country	Variable soil depth with high attenuation potential over groundwater with low attenuation potential. Largely vertical transport except when soils are saturated. Mostly overland flow pathway for variants.	Moderate, High (AD), Very High (OF)	0.35, 0.50(AD), 0.65(OF)
Central Plains	Poorly drained soils with high attenuation potential overlie groundwater with low attenuation potential. Transport pathway is temporally variable: vertical in summer due to soil cracking and horizontal in winter.	High	0.5
Gleyed	Poorly drained soils with high attenuation potential but dominantly horizontal drainage (naturally or by artificial drainage). All farmable land parcels are assumed to be artificially drained.	High, Very High (OF)	0.5, 0.65(OF)
Lignite/Marine Terraces	Well drained soils with dominantly vertical drainage except for variants when soils are saturated. High attenuation potential in groundwater.	Low, High (AD), Very High (OF)	0.2, 0.5(AD), 0.65(OF)
Old Mataura	Well drained soils with negligible attenuation potential over groundwater with low attenuation potential. Dominant drainage in vertical direction.	Very High	0.65
Oxidizing	Well drained soils with limited nitrogen attenuation potential over groundwater with low attenuation potential. Dominantly vertical drainage except for variants when soils are saturated.	High, High (AD), Very High (OF),	0.5, 0.5(AD), 0.65(OF)
Peat Wetlands	Poorly drained soils over ground water with high attenuation potential. Low attenuation due to dominantly horizontal drainage.	High	0.5
Riverine	Shallow well drained soils with negligible attenuation potential over groundwater with negligible attenuation potential. Dominant drainage in vertical direction.	Very High, Very High (OF)	0.65, 0.65(OF)

The combined reduction potential of soils and groundwater and dominant contaminant pathways are from Hughes et al. (2016). The first values in the susceptibility index column are for the physiographic zone and additional values are for the variants associated with artificial drainage (AD) and overland flow (OF).

The lowest nitrogen loss susceptibility is associated with the Lignite/Marine Terraces physiographic zone (Fig. 3) due to a dominance of vertical drainage and high denitrification in the groundwater. The highest nitrogen loss susceptibility is associated with the Alpine physiographic zone due to negligible reduction by shallow soils and horizontal transport. The Old Mataura and Riverine physiographic zones have very high susceptibility due to a lack of reduction potential in soils and groundwater as does the Gleyed physiographic zone due to largely horizontal drainage.

### Delivered Load and Accumulated Susceptibility

The land-water system model represents transport, accumulation, and attenuation of nitrogen losses from all land upstream of each receiving environment. Because deep groundwater systems were not explicitly represented, it was assumed that all groundwater re-emerges into the surface water within the sub-catchment in which the groundwater drainage occurs. Although this was a simplification, it was reasonable because Southland does not have large aquifer systems and groundwater transit times are generally short (Wilson 2011).

The transport and attenuation of scenario source loads to each downstream receiving environment was represented by the SPARROW model. As well as estimating the nitrogen loss rates from all non-farmed land, the SPARROW model represents nitrogen attenuation as a first-order decay rate that is applied along the transport path. The delivered load for each receiving environment (Fig. 2) was calculated as the sum of the attenuated individual scenario source loads from all upstream land parcels with units of kg TN year^−1^. The delivered load at a receiving environment was used to compute the pressure indicator as explained in the “PEC indicators” section.

The accumulated susceptibility (Fig. 2) associated with all land parcels upstream of each receiving environment was also based on the digital drainage network and represents an area weighted mean nitrogen loss susceptibility as follows:

1 $${\rm{Accumulated}}\,{\rm{susceptibility}} = \frac{{\mathop {\sum}\nolimits_{i = 1}^N {{\rm{Nitrogen}}\,{\rm{loss}}\,{\rm{susceptibility}}_i \times A_i} }}{{\mathop {\sum}\nolimits_{i = 1}^N {A_i} }}$$

where Nitrogen loss susceptibility_*i*_ is the loss susceptibility index for the *i*th land parcel, *N* is the total number of farmable land parcels upstream of a receiving environment, and *A*_*i*_ is the area of the *i*th land parcel. The accumulated susceptibility was calculated based only on the farmable land parcels so that it represents a catchment average susceptibility associated with land use and avoids incorporating land that cannot be used for production such as the conservation estate. The accumulated susceptibility at a receiving environment was used to compute the relative contribution indicator as explained in the “PEC indicators” section.

### Environmental Objectives and Maximum Acceptable Loads

Environmental objectives (Fig. 2) for freshwater receiving environments in New Zealand are mandated by the National Policy Statement for Freshwater Management (NPS-FM; NZ Government 2020) and for coastal receiving environments by the New Zealand Coastal Policy Statement (NZCPS; NZ Government 2010). Objectives that limit eutrophication of freshwater receiving environments are a specific requirement of the NPS-FM and are consistent with the objectives of NZCPS. We therefore used objectives for river and estuary receiving environments that were consistent with the requirements of the NPS-FM and NZCPS to define the environmental constraints in the PEC assessment.

Under the NPS-FM, water quality objectives for receiving environments must be set by decision makers at the regional level and must be at least at a predefined minimum acceptable state (see Supplementary Material for details). The NPS-FM requires that objectives to limit eutrophication of rivers are defined in terms of on periphyton (benthic algae) biomass thresholds and nitrogen concentration criteria must be set to achieve those objectives. Nitrogen concentration criteria as median values (mg TN m^−3^) to achieve periphyton biomass objectives consistent with the minimum acceptable state were obtained for each river receiving environment from Snelder et al. (2019). The criteria accounted for differences between receiving environments in factors that influence periphyton biomass, other than TN concentration, such as hydrological regime, light and temperature. The concentration criteria were converted to equivalent maximum acceptable loads (MAL; kg TN year^−1^) using the method of Snelder et al. (2020) (see Supplementary Material for details). The environmental constraint for each river receiving environment was therefore defined by a MAL to achieve a minimum acceptable state for periphyton biomass.

We used the New Zealand Estuary Trophic Index (ETI, Plew et al. 2020) to define environmental constraints for each estuary in the study area. The ETI quantifies TN loads (kg TN year^−1^) for estuaries that will achieve algal biomass objectives, which are analogous to the NPS-FM periphyton biomass bands. The loads are specific to each estuary and account for differences between estuaries in factors that influence algal biomass, other than TN load, such as morphological and hydrodynamic conditions (see supplementary material for details). The environmental constraint for each estuary receiving environment was therefore defined by a MAL to achieve a minimum acceptable state for algal biomass.

### PEC Indicators

The productive potential indicator (Fig. 2) for farmable land parcel in the study area was assigned to each land parcel by expressing its ordinal LUC class as one of three categories. High, medium and low productive potential were assigned to the LUC classes 1–3, 4–5 and 6–8, respectively.

The relative contribution and pressure indicators (Fig. 2) for a land parcel can be calculated for any downstream receiving environment. For each land parcel, we produce a single “most relevant” value of each indicator based on defining “critical points”. A critical point was defined for each land parcel as the downstream receiving environment with the highest ratio of the delivered scenario load to MAL. Individual catchments (i.e., defined by the entire drainage path upstream of a terminal segment) can have one critical point (the terminal segment) or multiple critical points, which include the terminal segment. Each land parcel therefore exists within one critical point catchment, which is defined by the downstream critical point.

The relative contribution indicator for each land parcel expresses the extent to which its nitrogen loss susceptibility is higher or lower than the average nitrogen loss susceptibility of all land parcels belonging to a critical point catchment. Relative contribution was calculated from the loss susceptibility indices of all individual land parcels in each critical point catchment as follows:

2 $${\rm{Relative}}\,{\rm{contribution}}_i = \frac{{{\rm{Nitrogen}}\,{\rm{loss}}\,{\rm{susceptibility}}_i \,-\, {\rm{Accumulated}}\,{\rm{susceptibility}}}}{{{\rm{Accumulated}}\,{\rm{susceptibility}}}}$$

where Nitrogen loss susceptibility_*i*_ and Accumulated susceptibility are as defined for Eq. (1).

Relative contribution is a continuous variable with most values in the range −3 to 3 when the nitrogen loss indices are normally distributed. Negative values indicate land parcels that have nitrogen loss susceptibility lower than the mean and vice versa. We converted these continuous values into three relative contribution indicator categories, (low, medium and high) with the subjective ranges −∞ to –0.5, −0.5 to 0.5, and 0.5 to +∞, respectively.

For each scenario, the pressure indicator for the land parcels upstream of each critical point was evaluated as the ratio of delivered load to MAL at the critical point. This value theoretically varies between 0 and +∞, with values larger than one indicating that delivered loads exceed the MAL. We used the terms excess and headroom to indicate receiving environments where delivered loads were greater than and less than the MAL, respectively. The continuous values were converted to the pressure indicator by expressing them as four categories; large headroom, small headroom, small excess, and large excess, defined by the values <0.5, 0.5–1, 1–10 and >10, respectively.

### LUS Index

The primary outputs of a PEC assessment are categorical values of the three indicators and maps showing the distribution of categories for each indicator across the assessment area. However, results of a PEC assessment could be reported and visualized as a single index calculated from the combined categories for all three indicators. We refer to this composite index as a “LUS index”. The LUS index is intended to group combinations of indicator categories that identify land parcels judged to have a similar level of suitability for agricultural production given a set of environmental constraints

Numerous quantitative and qualitative approaches have been used to develop composite indicators (e.g., Joint Research Centre-European Commission 2008). To provide a simple example, we used an additive, unweighted approach to derive an LUS index based on the PEC indicator categories in three steps. First, numeric values were assigned to the indicator categories. The productive potential categories low, medium and high were assigned 1, 3 and 5; the relative contribution categories high, medium and low were assigned 1, 3 and 5; and the four pressure categories large headroom to large excess were assigned 5, 4, 2 and 1, respectively. Second, for every combination of the three indicators (36 possible combinations), the numeric values were summed. Third, the resulting set of summed values were standardized to range between one and five. The distribution of these LUS index values across the PEC indicators is shown in Table 2.

**Table 2:** LUS index values for all possible combinations of categories of the productive potential, relative contribution and pressure indicators

	Productive potential								
	High			Medium			Low		
	Relative contribution								
Pressure	Low	Med	High	Low	Med	High	Low	Med	High
Large headroom	5	4	4	4	4	3	4	3	2
Small headroom	5	4	3	4	3	3	3	3	2
Small excess	4	3	3	3	3	2	3	2	1
Large excess	4	3	2	3	2	2	2	2	1

The standardized values range from 1 (least suitable) to 5 (most suitable) for agricultural production given environmental constraints

## Results

### Indicators and Response to Land-use Scenarios

**Fig. 4:**
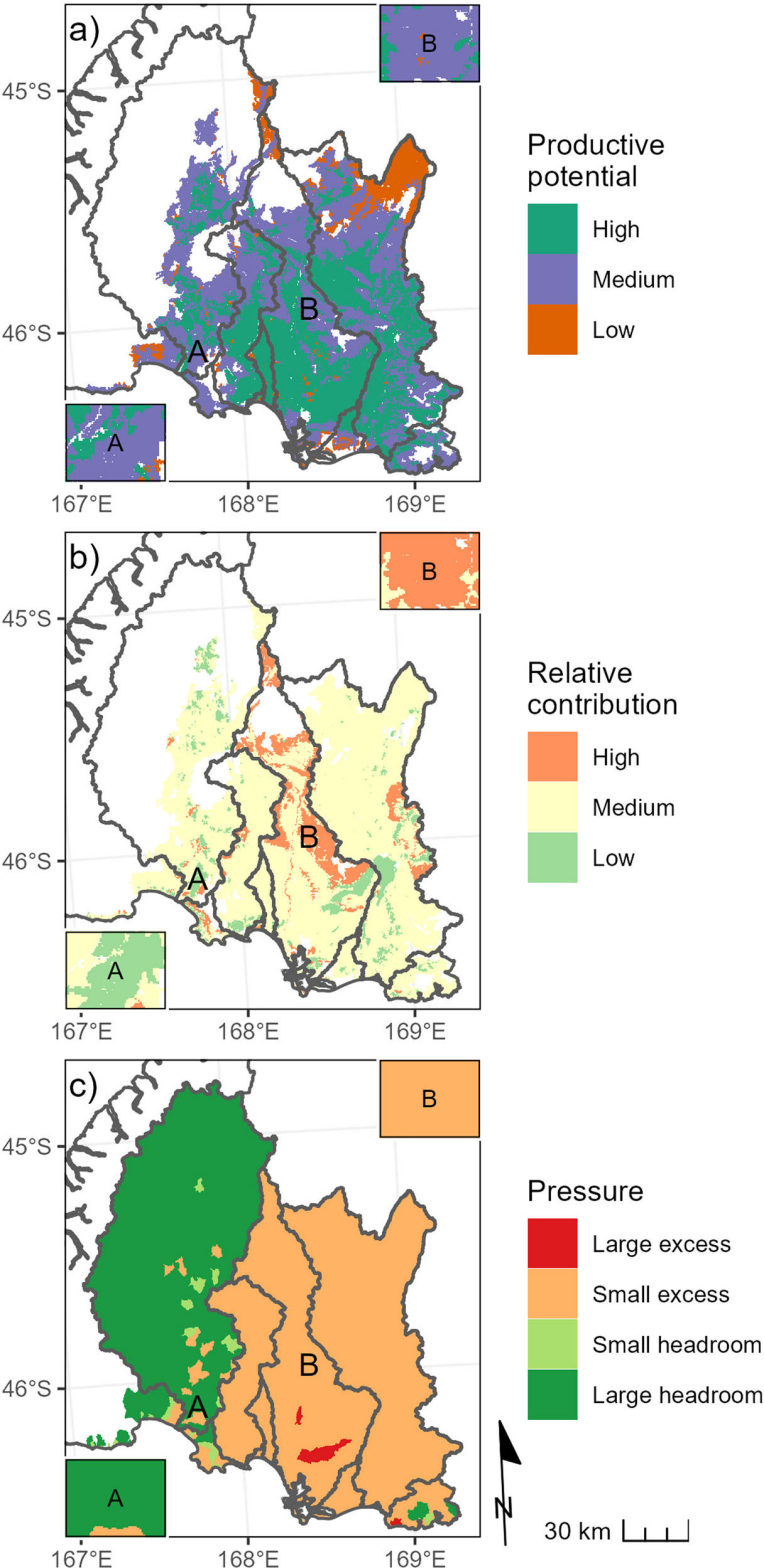
Mapped outputs of the PEC assessment for Southland, New Zealand. The results shown are for current land-use scenario and environmental constraints defined by the minimum acceptable state for all river and estuary receiving environments. The productive potential (**a**) and relative contribution (**b**) indicators are only evaluated and shown for farmable land parcels, while the pressure indicator (**c**) is evaluated and shown for the catchment upstream of critical points. The labels A and B on the maps are areas with generally contrasting combinations of PEC indicators. The inset boxes on each panel with the same labels are smaller scale maps of the areas approximately covered by the corresponding letters on the maps. The inset boxes represent an area with a width of 9 km

Results of the PEC assessment of Southland, based on the current land-use scenario and the environmental constraint defined by the minimum acceptable state for all river and estuary receiving environments, are shown in Fig. 4 and Table 3. Productive potential was highest in the low-elevation, low-gradient areas of the region’s major catchments. Farmable land parcels with low productive potential were mainly located in high-elevation locations. Productive potential was medium in most of the intermediate elevation land across the region and in some locations close to the coast (Fig. 4).

**Table 3:** Total areas and proportions of farmable land parcels in PEC indicator categories in Southland, New Zealand

Indicator	Category	Current land-use scenario		Maximum land-use scenario	
		Total area (ha)	Proportion of farmable land parcels (%)	Total area (ha)	Proportion of farmable land parcels (%)
Productive potential	High	549,493	43.3	549,493	43.3
	Medium	614,932	48.5	614,932	48.5
	Low	104,224	8.2	104,224	8.2
Relative contribution	High	93,658	7.4	92,711	7.3
	Medium	1,050,316	82.5	1,050,026	82.5
	Low	129,328	10.2	130,565	10.3
Pressure	Large excess	14,904	1.2	76,907	6.0
	Small excess	1,036,742	80.5	988,792	76.8
	Small headroom	24,262	1.9	205,942	16.0
	Large headroom	211,619	16.4	15,885	1.2

The values are for an assessment based on the environmental constraint defined by the minimum acceptable state for all river and estuary receiving environments and the current and maximum land-use scenarios

The pressure category was small excess across most of the Aparima, Ōreti and Mataura River catchments (Fig. 4; see Fig. 1 for place names). The pressure category was large excess for land parcels in two sub-catchments of the Ōreti River catchment, indicating that the delivered loads at critical points exceeded the MAL by at least 10 times. The pressure category varied across sub-catchments of the Waiau River catchment, with most parcels in the large headroom category, but some small sub-catchments in the small excess category. In part, sub-catchments in the large headroom pressure category in the Waiau River catchment reflect the low accumulated loads throughout the catchment due to the relatively small proportion of farmable land parcels compared to other catchments. Because the accumulated load to the Waiau River estuary is less than the MAL, sub-catchments in the excess pressure categories are governed by critical points that are located upstream of the estuary and are associated with river segments that have TN loads in excess of their MALs (Fig. 4).

Approximately 73% of the farmable land in Southland that we classified as high productive potential (LUC classes 1–3) was used for intensive sheep, beef and deer farming, and ~23% was used for dairy farming. At the region-wide-scale, shifting from the current land-use scenario to the maximum pastoral land-use scenario increased the proportion of farmable land parcels in the large excess pressure category from 1.2 to 6.0% and reduced the combined proportion of farmable land parcels in the small and large headroom categories from 18.3 to 17.2% (Table 3).

**Fig. 5:**
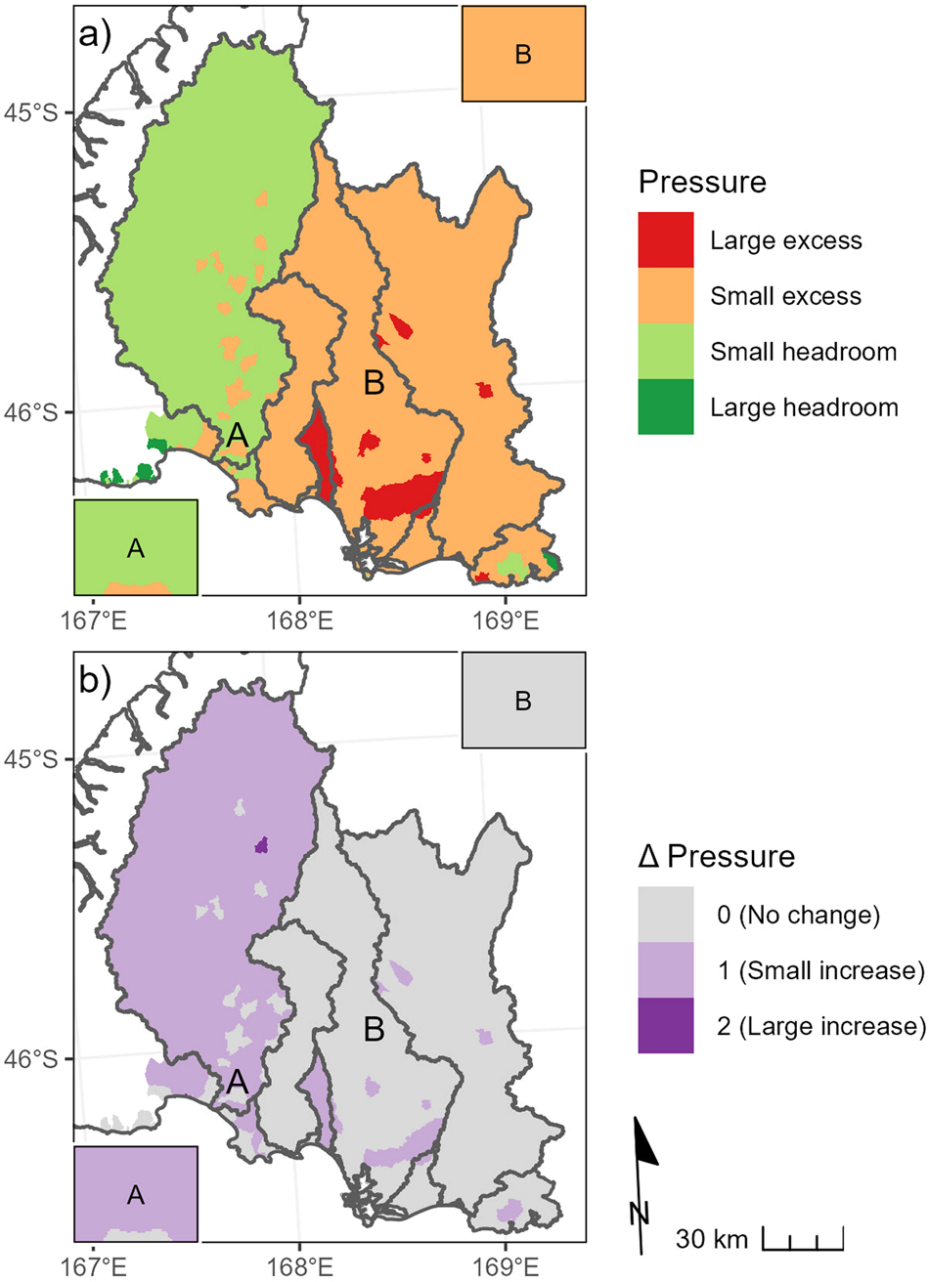
Map of the pressure indicator for the maximum land-use scenario and environmental constraints defined by the minimum acceptable state for all river and estuary receiving environments (**a**) and the difference in the pressure categories between the maximum and current land-use scenarios (**b**). Small and large pressure change on map (**b**) indicates a change of one and two pressure categories on the scale shown in map (**a**), respectively The pressure indicator is shown for the entire catchment upstream of critical points for catchments that contain some farmable land parcels in the region. See caption of Fig. 4 for explanation of the labels A and B on the maps and the inset boxes on each panel

Large increases in delivered scenario loads under the maximum land-use scenario caused changes in the locations of some critical points and changes in pressure categories for some land parcels. The pressure category was generally higher for land parcels in the Waiau River catchment under the maximum land-use scenario compared to the current land-use scenario (Fig. 5). For most land parcels in the Aparima, Ōreti and Mataura River catchments, the pressure category did not change between the current and maximum land-use scenarios. However, there were changes from the small to large excess category for land parcels in some sub-catchments of the Aparima, Ōreti and Mataura Rivers (Fig. 5).

For the current land-use scenario and the environmental constraint defined by the minimum acceptable state, the relative contribution category was medium for most land parcels in the region (Fig. 4). Because the relative contribution indicator is based on comparisons of land upstream of a critical point, land parcels with the same nitrogen loss susceptibility but located in different catchments can have different relative contribution values. Shifting from the current land-use scenario to the maximum land-use scenario made little difference to the proportions of farmable land parcels in the three relative contribution categories at the region-wide-scale (Table 3).

### Using the PEC Indicators to Characterize Land Use Suitability

Two areas with contrasting categories for two of the three PEC indicators are marked A and B on Fig. 4. Land parcels in area A were in the medium category for productive potential, the large headroom category for pressure, and the low category for relative contribution. Land parcels in area B were in the medium category for productive potential, the small excess category for pressure and the high category for relative contribution.

Given that land parcels in areas A and B had the same productive potential, it is reasonable to consider area A to be more suitable for intensive land use with the potential for high losses of nitrogen than area B. However, judging the relative suitability of areas A and B would be more complicated if the relative contribution or pressure categories in areas A and B were reversed (e.g., if areas A and B had high and low relative contribution categories, respectively). In this case it would be less obvious which area is more suitable because it depends on the weight given to relative contribution and pressure in the overall judgment of suitability.

**Fig. 6:**
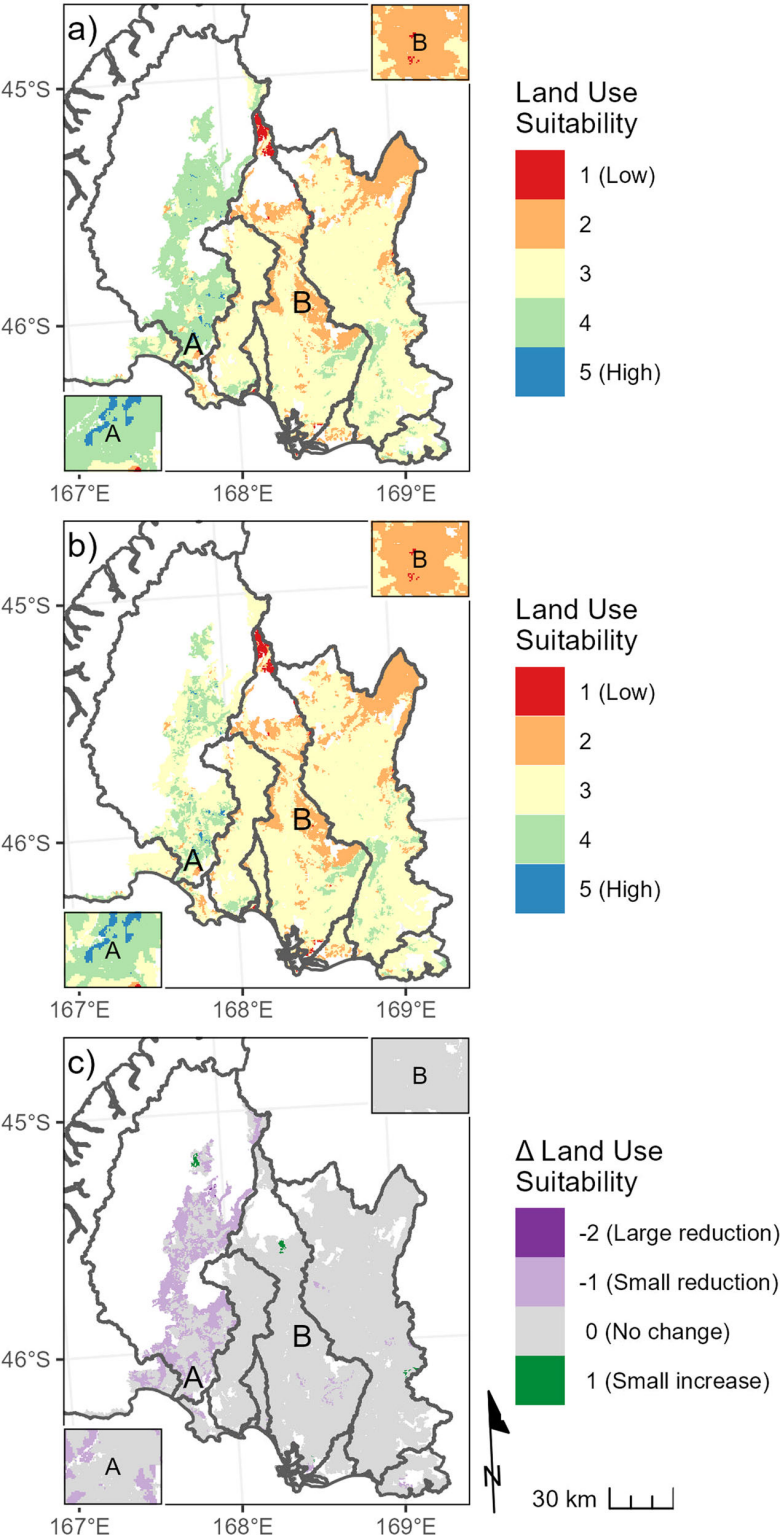
Mapped LUS index showing suitability for agricultural production given a set of environmental constraints under the current (**a**) and maximum (**b**) land-use scenarios and the difference between the two (**c**). For both scenarios, the pressure indicator was evaluated for the environmental constraint defined by the minimum acceptable state for all river and estuary receiving environments. The change in land use suitability on map (**c**) indicates a change of one and two of the categories on the scale shown in map (**a**), respectively. See caption of Fig. 4 for explanation of the labels A and B on the maps and the inset boxes on each panel

When the scheme for combining and weighting the indicators (Table 2) was applied to the PEC indicators representing the current land-use scenario, much of the low-elevation, low-gradient areas of the major catchments (comprising most of the Southland plains) had the intermediate LUS index value of three (Fig. 6). Compared to the rest of the study area, a higher proportion of land parcels in the Waiau River catchment had LUS index values of four under the current land-use scenario (Fig. 6a). This reflects the predominance of the large-headroom pressure category in this catchment. Under the maximum land-use scenario, pressure increased in parts of the Waiau catchment, and the LUS index for many land parcels decreased from four to three (Fig. 6c).

The difference between maps (a) and (b) (shown by c) in Fig. 6 emphasizes that LUS index values reflect choices made in the PEC assessment. Differences in the LUS index values for land parcel between maps (a) and (b) in Fig. 6 are associated with differences in the land-use scenarios used to define each map. Differences in the LUS index values would also occur if different environmental constraints were chosen (e.g., if objectives were more stringent than the minimum acceptable state). The LUS index is also dependent on the way the assessed PEC indicator categories are combined; other formulations of the scheme shown in Table 2 would produce different spatial patterns.

## Discussion

### Land Use Suitability Information for Decision-Making

Decisions about environmentally sustainable land use require information that links agricultural production to the potential of land, and information about impacts on other aspects of the larger land-water system in which a land parcel is embedded (McDowell et al. 2018). These decisions need to be supported by assessments of the cumulative impacts of land use on multiple values across broad areas. A PEC assessment is an example of this type of assessment, narrowly focused on cumulative impact of land use on the risk of eutrophication of downstream receiving environments. PEC indicators can be used either individually or collectively (e.g., as the LUS index described in the “LUS index” section) to assist in land-use decision-making and management.

In this study, the productive potential indicator identified that there is considerable potential for land-use intensification in the Southland region. However, the pressure indicator identified that current nitrogen loads are generally unacceptably high and that nitrogen targets for rivers and estuaries are already being exceeded. This result is consistent with the findings of Snelder et al. (2020) who concluded that nitrogen discharges to aquatic receiving environments in the Southland region frequently exceed New Zealand’s minimum environmental criteria. Further intensification of land use would increase the risk to downstream receiving environments. Correspondingly, the land identified as most suitable for increased production are those parcels with high productive potential that are assigned to the headroom pressure and low relative contribution categories (Fig. 6).

The information provided by PEC assessments can be used in land-management decision-making processes that are intended to promote shifts in agricultural production toward more suitable locations or to formulate interventions to reduce impacts. Specific applications may focus on different PEC indicators and/or categories. For example, land investors can use PEC indicators to identify land with the highest suitability for land-use intensification (i.e., high productive potential, low relative contribution, located in catchments with headroom). Strategies aimed at reducing current contaminant losses can use PEC indicators to identify land parcels where mitigations (e.g., changing or avoiding particular management practices) will have the greatest benefits, such as land parcels with high relative contribution located in catchments with excess contaminant loads.

### Subjectivity in Characterizing Land Use Suitability

The three PEC indicators provide a basis for characterizing the relative suitability of land parcels for land uses that result in nitrogen losses. It is important to acknowledge that characterizations of LUS entail several subjective choices (Fig. 1). We distinguish two general types of choices; those that concern how indicators are evaluated, and those that concern how indicators are combined and weighted to judge LUS.

Evaluations of the relative contribution and pressure indicators are influenced by choices of land-use scenarios and environmental constraints. Choices of land-use scenarios influence the delivered loads and therefore the relative contribution indicator. Choices of environmental constraints influence the pressure indicator. In practice, choices of environmental constraints will be influenced by existing policies or regulations. In New Zealand, for example, the NPS-FM prohibits setting environmental objectives for rivers and estuaries that are less stringent than the minimum acceptable state, but communities can increase the stringency of those objectives. The sensitivity of PEC indicators to choices of land-use scenarios and environmental constraints, and the subsequent judgments about suitability reflect the normative nature of environmental objective setting (Slocombe 1993; Grumbine 1994).

PEC assessments also involve choices about how the indicators are combined and weighted irrespective of whether an LUS index is derived or judgments about suitability are made based on the separate indicators. When comparing two land parcels, judgments about relative suitability may be reasonably clear from inspection of the individual indicator categories, such as in the example shown on Fig. 4. However, the same example shows that judgments are not always straightforward because they involve trade-offs between competing objectives (i.e., agricultural production versus environmental impacts).

Other studies have linked broadscale land use options to receiving environment impacts to produce single, definitive LUS maps. Two methods used to produce such maps are spatial optimization (e.g., Xu et al. 2018) and multi-criteria analysis (e.g., Waltham et al. 2021). Both methods involve steps that determine an acceptable trade-off between agricultural production and environmental impacts. These steps involve subjective decisions that are therefore implicit in the resulting LUS maps. Conveying the results of PEC assessments as maps of three independent indicators is less specific and less directive than a single, definitive LUS map, and the former approach requires the user to explicitly consider the trade-offs.

There may be situations in which a PEC assessment is best reported and visualized using a single composite index. However, defining the composite index involves making choices about how the indicators should be combined and weighted, and these choices reflect judgments about how to achieve an acceptable balance between agricultural production and environmental impacts.

Our PEC assessment of the Southland region makes it clear that LUS is not an intrinsic characteristic of a land parcel but depends on multiple subjective decisions. In addition, because consideration of the land-water system in which a land parcel is embedded is a fundamental tenet of the LUS concept, a PEC assessment for any land parcel depends on land use elsewhere in the catchment and this can change independently of the land parcel being considered.

### Accurate Representation of the Land-Water System

Our land-water system model of Southland represented complex biophysical processes using the best available models. However, all component models were simplified representations of reality with limited spatial detail. The representation of two sets of processes in particular, attenuation of nitrogen lost from land and receiving environment responses to nitrogen inputs, impacted on the resolution and accuracy of the PEC assessment.

The SPARROW model, which was used to calculate nitrogen loads delivered to each receiving environment, represents attenuation as a first-order decay function of distance traveled in stream channels (Alexander et al. 2004; Elliott et al. 2005). This representation does not account for fine-scaled variation in soil and groundwater redox conditions that produce spatially variable rates of attenuation (Rivett et al. 2008; Landon et al. 2011). Currently, the best description of spatial variation in attenuation associated with soil and groundwater redox conditions in Southland is the physiographic classification (Hughes et al. 2016). Because land parcels in our land-water system model had a characteristic scale of 66 ha, we used the relatively fine-scaled physiographic information to evaluate the susceptibility of land parcels to nitrogen loss and relative contribution at the land parcel scale. However, the physiographic classification represents nitrogen loss susceptibility within the footprint of the land parcel and does not account for any attenuation occurring in the drainage network downstream of the land parcel. Therefore, different approaches were used to represent attenuation to make the best use of the available knowledge and tools that are relevant to the two indicators but both representations had limited accuracy.

Conceptually, improved representation of both delivered loads and nitrogen loss susceptibility could be achieved by modeling nitrogen mobilization, transport and attenuation at finer spatial scales. However, prospects for fine-scale models are limited by our understanding of attenuation processes and the availability of data, including for model calibration (i.e., locations with measured nitrogen loads, measurements of attenuation). We used an expert assessment of nitrogen loss susceptibility for physiographic zone and variants in the absence of a suitable numerical model. The relative contribution indicator depends on the nitrogen loss susceptibility index; therefore, the susceptibility indices are a potential source of inaccuracy in our assessment.

The spatial detail and accuracy of the land-water system model was also limited by our ability to predict responses by algae to nitrogen inputs in individual rivers and estuaries. The environmental constraints (i.e., MALs) for rivers and estuaries were derived from models that account for variation in the large-scale drivers of trophic response. However, these models have high site-scale uncertainty (Snelder et al. 2019; Plew et al. 2020). Because the MAL was evaluated for individual receiving environments, there was uncertainty associated with the identification of critical points and subsequent assignment of pressure categories to land parcels.

Similar limitations to resolution and accuracy of land-water system models can be expected in any implementation of the LUS approach. Therefore, use of outputs of LUS assessments to support land use decision making needs to be at scales and levels of detail that are appropriate to the resolution and accuracy of the assessment. The limitations in the representation of nitrogen attenuation and receiving environment responses in our study mean the outputs have utility for broadscale applications such as regional land use planning but may not be sufficiently accurate for applications that involve decision making at the scale of individual land parcels.

## Conclusions

This study extended the traditional assessment of the suitability of land within a farm boundary for agricultural production to include consideration of environmental constraints defined by water-quality objectives for downstream receiving environments. We used existing tools and models to carry out an assessment of LUS in a region of New Zealand, but the assessment was limited by the spatial resolution and accuracy of the data and the ability to represent the relevant processes.

Our study highlights the facts that LUS is inherently subjective and is not an intrinsic property of a land parcel. An assessment of suitability of an individual land parcel for a given land use is dependent on choices about land use elsewhere in the landscape, the environmental objectives that determine the constraints, and how the indicators are combined and weighted. Understanding how subjective decisions influence the assessment of LUS would be aided by allowing users and decision makers to rerun assessments using different sets of choices.

The plurality of potential results of PEC assessments indicates that they should be presented using an interactive system, preferably an interactive GIS that allows the indicators and the underlying data to be inspected and combined in alternative ways. Such a tool would allow environmental managers to test different options and demonstrate to land users why land-use choices should not be determined by potential productivity alone but should also account for environmental constraints and for land use elsewhere in the landscape.

## Supplementary information

Supplementary Information
