# Supplementary material for: Land-use Suitability is Not an Intrinsic Property of a Land Parcel
Source: Environ Manage. 2022 Dec 16;71(5):981–97. doi: 10.1007/s00267-022-01764-y (PMC10083159; doi:10.1007/s00267-022-01764-y)
Supplement: Supplementary file 1 — Supplementary Information [file 267_2022_1764_MOESM1_ESM.docx]

# Supplementary material - Land-use suitability is not an intrinsic property of a land parcel.

This supplementary material contains details of some aspects of the analyses used to model the land-water system in the Southland study area. These aspects include:

- the land cover categories,
- nutrient criteria for rivers and estuaries,
- conversion of river nutrient concentration criteria to maximum allowable loads.

# Land cover categories

Land was assigned to one of three land cover categories (agricultural, urban and natural) based on a reclassification of classes defined by the national land cover database version 4 (LCDB4; lris.scinfo.org.nz). LCDB4 differentiates 33 land-cover categories based on the Satellite Pour l'Observation de la Terre (SPOT-5) imagery from 2012. The reclassification of the LCDB4 classes into the land cover categories used by this study is shown in Table 1. Note that land that is categorized as Agricultural is further classified into farm types based on combinations of four factors: enterprise type (dairy, sheep and beef, forestry); 2) land-use intensity level (high, medium, low); 3) land-use capability (LUC) category (1 - 8); and 4) drainage type (well drained, poorly drained).

Table 1. Reclassification of the 33 LCDB4 classes into land cover types used by this study

| **Land Cover Group** | **LCDB4 class** | **Class Name** | **Land cover category: this study** |
| --- | --- | --- | --- |
| Artificial Surfaces | 1 | Built-up Area (settlement) | Urban |
|  | 2 | Urban Parkland/Open Space | Urban |
|  | 5 | Transport Infrastructure | Urban |
|  | 6 | Surface Mine or Dump | Urban |
| Bare or lightly-vegetated Surfaces | 10 | Sand or Gravel | Natural |
|  | 12 | Landslide | Natural |
|  | 14 | Permanent Snow and Ice | Natural |
|  | 15 | Alpine Grass/Herbfield | Natural |
|  | 16 | Gravel and Rock | Natural |
| Water bodies | 20 | Lake or Pond | Natural |
|  | 21 | River | Natural |
|  | 22 | Estuarine Open Water | Natural |
| Cropland | 30 | Short-rotation Cropland | Agricultural |
|  | 33 | Orchard, Vineyard or Other Perennial Crop | Agricultural |
| Grassland, Sedgeland and Marshland | 40 | High Producing Exotic Grassland | Agricultural |
|  | 41 | Low Producing Grassland | Agricultural |
|  | 43 | Tall Tussock Grassland | Agricultural |
|  | 44 | Depleted Grassland | Agricultural |
|  | 45 | Herbaceous Freshwater Vegetation | Natural |
|  | 46 | Herbaceous Saline Vegetation | Natural |
|  | 47 | Flaxland | Natural |
| Scrub and Shrubland | 50 | Fernland | Natural |
|  | 51 | Gorse and/or Broom | Agricultural |
|  | 52 | Manuka and/or Kanuka | Natural |
|  | 54 | Broadleaved Indigenous Hardwoods | Natural |
|  | 55 | Sub Alpine Shrubland | Natural |
|  | 56 | Mixed Exotic Shrubland | Agricultural |
|  | 58 | Matagouri or Grey Scrub | Natural |
| Forest | 64 | Forest - Harvested | Agricultural |
|  | 68 | Deciduous Hardwoods | Agricultural |
|  | 69 | Indigenous Forest | Natural |
|  | 70 | Mangrove | Natural |
|  | 71 | Exotic Forest | Agricultural |

# Nutrient criteria for rivers

The National Policy Statement for Freshwater Management (NPS-FM; NZ Government 2020) includes the National Objectives Framework (NOF), which specifies ‘attributes’ (water quality indicators) that regional councils (regional regulatory authorities in New Zealand) must use to define numeric objectives for water quality. Attributes provide a graduated range of stringency consisting of A, B, C and D ‘bands’ where A indicates near natural condition and the D-band representing a state that is unacceptable in all rivers. Choosing water quality objectives from the acceptable bands (A to C, where C is the minimal acceptable state) is the responsibility of regional councils and must consider the impact on social, cultural, economic and/or ecological values.

With respect to nitrogen and rivers, the NOF defines three relevant attributes; periphyton biomass (algae attached to the surfaces of riverbeds), and nitrate and ammonia toxicity. Eutrophication of rivers due to enrichment by nutrients, primarily nitrogen and phosphorus, is a significant environmental issue in New Zealand (Biggs and Price 1987). The periphyton attribute is used to define trophic state objectives for rivers based on a limiting algal biomass. The NPS-FM requires that water quality objectives for periphyton biomass are set for all streams and rivers by choosing from the A, B or C band, which are defined by algal biomass thresholds of 50 mg m^-2^, 120 mg m^-2^, 200 mg m^-2^ chlorophyll *a*, respectively. To achieve the chosen objective, periphyton biomass must be less than the threshold for 11 out of 12 monthly measurements on average (NZ Government 2020). The NPS-FM also requires that regional councils set nutrient concentration criteria that will ensure that periphyton objectives are achieved and undertake management actions to prevent or reduce elevated nutrients in situations where criteria are exceeded.

Nitrate and ammonia are toxic at concentrations that are generally higher than those required restrict periphyton biomass. Because streams in Southland have habitat that supports periphyton, instream nitrogen must generally be managed to achieve the more restrictive periphyton attribute, rather than the toxicity attributes. Therefore, our PEC assessment was based on river nitrogen concentration criteria for achieving nominated periphyton bands, and not to restrict nitrate and ammonium toxicity. The nitrogen concentration criteria we used were specific to the NOF A, B and C bands for periphyton and are based on total nitrogen (TN).



Snelder et al. (2019) derived total nitrogen (TN) concentration criteria that achieve periphyton biomass objectives defined by the three attribute bands (A, B and C). These criteria are defined by median TN concentrations and were derived using a regression model fitted to a dataset comprising 20 years of monthly measurements of periphyton biomass and environmental predictor variables at 78 monitoring sites across New Zealand. The environmental predictor variables account for broad-scale variation in physical conditions that influence the biomass other than nutrient concentrations (e.g., temperature, light regimes and frequency of high flows that remove periphyton from the riverbed).

Separate TN concentration criteria were derived for each of 21 river classes defined by the second level of the New Zealand River Environment Classification (REC; Snelder and Biggs 2002). REC classes at the second level of the REC broadly distinguish between rivers based on climate and topography and classes and these discriminate variation in factors that influence the biomass response to nutrient supply other than nutrient concentrations. The concentration criteria for each REC class are therefore specific to the physical conditions that can be expected to influence periphyton biomass. The TN concentration criteria corresponding to each NOF band was obtained for each river receiving environment based on its REC class. REC class assignments were obtained from data that is associated with the digital river network.

# Nutrient criteria for estuaries

Environmental objectives for estuaries and other coastal environments are mandated by the New Zealand Coastal Policy Statement (NZCPS; NZ Government 2010). The NZCPS does not have the structured approach based on attributes for defining water quality objectives of the NPS-FM. However, the NZCPS does require regional councils to seek to preserve the natural character of the coastal environment including its ecosystems and biodiversity, which requires the management of discharges to, and concentration of contaminants in, the coastal water bodies.

A large proportion of Southland estuaries in agriculture-dominated catchments are shallow and have extensive intertidal areas. When enriched with nutrients (particularly nitrogen) from the upstream catchment, these estuaries are susceptible to eutrophication (Valiela et al. 1997; Viaroli et al. 2008). Symptoms of eutrophication in shallow estuaries include proliferations of benthic macroalgae, reduced oxygen levels, and sulfide production due to organic matter breakdown (Plew et al. 2020). The remaining Southland estuaries are deeper and predominantly subtidal. In deep estuaries, phytoplankton proliferations are symptomatic of eutrophication, rather than macroalgae. Most of the deep estuaries in Southland have catchments with little or no potential for pastoral land use and are therefore at low risk of eutrophication.

We used the New Zealand Estuary Trophic Index (ETI, Plew et al. 2020) to define TN targets for the Southland estuaries. The ETI quantifies the susceptibility of estuaries to eutrophication, based on their morphological characteristics and catchment TN load. The method predicts the potential in-estuary TN concentration by combining the catchment and oceanic contributions of TN (as annual catchment loads and mean annual ocean nutrient concentration) within an estuary dilution model that incorporates data describing estuary volume, tidal prism and freshwater and oceanic inflows (Plew et al. 2018). For each estuary in the region, the dilution model was used to predict the potential TN concentration corresponding to a range of catchment TN loads. The concentrations are used to define eutrophication susceptibility as macroalgal and phytoplankton response bands (Plew et al. 2020)that are analogous to the NOF river periphyton bands (i.e., A band indicating very low susceptibility and the D band indicating high susceptibility). For estuaries that have an intertidal area greater that 40%, ETI susceptibility bands are based on macroalgal biomass. For estuaries with intertidal areas less than 5%, ETI susceptibility bands are based on phytoplankton biomass. If the intertidal area is between 5% and 40%, ETI susceptibility bands are defined based on both macroalgal and phytoplankton biomass. Maximum acceptable loads for estuaries were defined as the loads corresponding to the threshold between the C and D bands for either macroalgal or phytoplankton response, depending on intertidal area.

# Conversion of river nutrient concentration criteria to maximum allowable loads

River TN concentration criteria were converted to equivalent maximum acceptable loads (MAL; kg TN year^-1^) using the method of Snelder et al. (2020) in five steps. First, site median concentrations were calculated from monthly observations of TN concentrations at 678 monitoring sites distributed throughout New Zealand for the five-year period ending 2017. The monitoring sites provided a reasonable representation of national variation in catchment characteristics such as climate, topography, geology and land use. The Southland Region was represented by 58 sites. Second, a Random Forest (RF) regression model (Cutler et al. 2007) was fitted to the site median TN values using the methods and predictor variables used by Snelder et al. (2020). The model predictors were catchment characteristics that are available for all segments of the digital surface water drainage network. Third, the fitted regression model was used to predict the current median TN concentration for every river receiving environment. Forth, we calculated the ratio of the current delivered load (i.e., for the current land use scenario) to the predicted TN concentration. Fifth, we multiplied the TN concentration criteria for each receiving environment by this ratio to derive the corresponding MAL for TN.

# References

Biggs BJF, Price GM (1987) A survey of filamentous algal proliferations in New Zealand rivers. New Zealand Journal of Marine and Freshwater Research 21:175–191

Cutler DR, Edwards JTC, Beard KH, et al (2007) Random forests for classification in ecology. Ecology 88:2783–2792

NZ Government (2020) National Policy Statement for Freshwater Management 2020

NZ Government (2010) New Zealand Coastal Policy Statement.

Plew DR, Zeldis JR, Dudley BD, et al (2020) Assessing the Eutrophic Susceptibility of New Zealand Estuaries. Estuaries and Coasts 43:2015–2033. https://doi.org/10.1007/s12237-020-00729-w

Plew DR, Zeldis JR, Shankar U, Elliott AH (2018) Using simple dilution models to predict New Zealand estuarine water quality. Estuaries and coasts 41:1643–1659

Snelder TH, Biggs BJF (2002) Multi-scale river environment classification for water resources management. Journal of the American Water Resources Association 38:1225–1240

Snelder TH, Moore C, Kilroy C (2019) Nutrient Concentration Targets to Achieve Periphyton Biomass Objectives Incorporating Uncertainties. JAWRA Journal of the American Water Resources Association 55:1443–1463

Snelder TH, Whitehead AL, Fraser C, et al (2020) Nitrogen loads to New Zealand aquatic receiving environments: comparison with regulatory criteria. New Zealand Journal of Marine and Freshwater Research 54:527–550

Valiela I, McClelland J, Hauxwell J, et al (1997) Macroalgal blooms in shallow estuaries: controls and ecophysiological and ecosystem consequences. Limnology and oceanography 42:1105–1118

Viaroli P, Bartoli M, Giordani G, et al (2008) Community shifts, alternative stable states, biogeochemical controls and feedbacks in eutrophic coastal lagoons: a brief overview. Aquatic Conservation: Marine and Freshwater Ecosystems 18:S105–S117
